# Hepatitis C virus infections and associated risk factors in patients with diabetes mellitus; case control study in North West Tigray, Ethiopia

**DOI:** 10.1186/s13104-018-3983-4

**Published:** 2018-12-10

**Authors:** Gebretsakan Gebrekristos, Mebrahtu Teweldemedhin, Letebrhan Hagos, Tuom Gebrewahid, Berihu Gidey, Hailay Gebreyesus

**Affiliations:** 1 Department of Medical Laboratory Sciences, College of Health Sciences, Aksum University, P.O. Box 298, Aksum, Tigray Ethiopia; 2 Department of Public Health, College of Health Science, Aksum University, Aksum, Tigray Ethiopia

**Keywords:** Hepatitis C virus, Prevalence, Association, Diabetes mellitus, Control group

## Abstract

**Objective:**

The objective of this study was to determine the seroprevalence of Hepatitis C virus among patients with Diabetes mellitus and healthy control groups in North West Tigray. Blood samples from each study subject was tested for Hepatitis C virus by using anti Hepatitis C virus antibody rapid test kits and confirmed using enzyme linked immuno sorbent assy.

**Result:**

The overall seroprevalence of Hepatitis C virus, Hepatitis C virus among diabetic and non diabetic study subjects were found (16.7, 28, and 6) % respectively. Multi varate logistic regression analysis result shows that study subject with uvulotomy, previous history of immunosuppressive disease, and study subjects with fast blood glucose (≥ 126 mg/dl) showed statistically significant association with anti Hepatitis C virus antibody sero status [AOR (12.4 (3.5–18.3); 0.1 (0.03–0.5); and 8.6 (1.7–13)] respectively.

## Introduction

Hepatitis C virus (HCV) infection and diabetes mellitus (DM) are two major public health problems that cause devastating health and financial burdens worldwide [1, 2]. Approximately 130–170 million are chronically infected with HCV, > 350,000 deaths/year [3]. Hepatitis C virus (HCV) infection is a frequent cause of acute and chronic hepatitis, and leads to the development of cirrhosis and hepatocellular carcinoma [3].

Infection with HCV has been shown to produce both hepatic and extrahepatic manifestations, the latter including insulin resistance, essential mixed cryoglobulinemia, and glomerulonephritis [4]. Cohort study in HCV infected patients indicated that, two-thirds of them experienced extra hepatic manifestations [5]. Soon after HCV discovery, HCV-related autoimmune or lymphoproliferative disorders, from benign mixed cryoglobulinemia to frank lymphomas, have been reported [4, 6]. HCV infection showed a higher mortality rate for extrahepatic complications [7–9]. All-cause mortality in patients with HCV was increased more than twice compared with patients without HCV [10]. It is evidenced that HCV contributes for the pathogenesis insulin resistance (IR). HCV attributed liver disease varies in spectrum and severity with potential end stage manifestations [5]. End stage liver diseases depend on several cofactors, mostly host-related cofactors, such as age, sex, level of alcohol consumption, overweight, immune status and co-infections [11, 12]. One of these cofactors is type 2 diabetes (T2D), which has been recognized to modify the course of hepatitis C even at the stage of insulin resistance (IR), a condition that precedes the development of T2D [13, 14]. A meta-analysis showed that HCV increases the risk of type 2 diabetes mellitus (T2DM) by 1.8 times in excess of that posed by relative degree of liver pathology [15].

The etiology of type II diabetes is not well known. But, recent studies showed that HCV infection could be associated with type II diabetes beyond to genetic, biologic, and demographic factors directly via glucose homeostasis perturbation of glucose metabolism; or indirectly through cytokine stimulation. Apart from this, HCV infection induces cytotoxic T cell response which damages hepatocytes [16, 17]. The contribution of HCV to the development of diabetes is explained by immunologic attack of the β-cell of the pancreas mainly due to the presence of HCV-RNA in the acinar cells and the epithelial lining of the pancreatic duct [18, 19].

Currently many studies establish the association of HCV and DM in a number of clinical studies though conflicting results are reported and interestingly, some studies have also shown that the prevalence of HCV infection in diabetics is much higher compared with the normal population [20–22]. Even though understanding the rate of their co-infection is important for appropriate medical management, continuous monitoring of liver function test and glucose abnormality, and giving awareness about the risk of transmission for HCV there is paucity of scientific data on the prevalence of HCV among diabetic patients in Ethiopia. Thus the objective of this study is to determine the prevalence HCV and associated risk factors among DM and control groups.

## Main text

### Methods

#### Study area, sample size and sampling techniques

The study was conducted at Suhul Zonal Hospital which is found north western zone of Tigray region. The hospital serves for greater than 1.2 million people including those who came from refugee camp. The zone is located at 1087 Kilometer from Addis Ababa, the capital city of Ethiopia. According to the Census conducted in 2007 North West zone has a total population of 736, 80 [23]. The sample size was calculated using a double proportion formula by considering the prevalence of hepatitis C virus in diabetic patients (p1) and non diabetic control group (p2), 9.9% vs 3.3%, respectively from previous study [21]. A total of 460 study participants were enrolled in this study by considering 95% confidence interval and 5% contingency for non responder.

#### Study design and setting

Health facility based case–control study was conducted from February to July 2017. Study was started by grouping the study subjects into two groups based on their exposure status i.e., diabetes and healthy control groups (blood donors and VCT service clients). The source population comprised all diabetic cases; blood donors and VCT service clients who attend their case in Suhul Hospital. The study population was those diabetic patients with age between 18 and 60 years, and blood donors and VCT service clients attending at Suhul Hospital during the study period.

#### Specimen management and laboratory test

Whole blood sample (5–10 ml) was collected aseptically from study participants. Plasma was separated as soon as possible to avoid hemolysis and only clear none hemolyzed specimen was used. Whole blood sample was used to measure Random blood sugar for the control groups. Plasma samples collected aseptically from each study subject was screened for anti HCV antibody using anti HCV detecting rapid test kits. Samples were stored at appropriate temperature and confirmed by Enzyme-Linked Immunosorbent Assay (ELISA). Positive results were communicated to respective clinician for further investigation and better management of clients.

#### Data processing and management

After taking informed consent from study participants’ relevant data for potential risk factors, socio demographic variables and other relevant information was collected using pre-tested structured questionnaire and Medical records were reviewed to get relevant clinical history. Laboratory result was recorded in the laboratory data report format. Questionnaire and laboratory data report format was checked for its completeness.

#### Data quality assurance

Prior to the actual work data collectors were trained how to go through consent form and questioner and questioner was pre tested for assuring clearness and understandability. Laboratory analyses were carried out using standard operating procedures (SOPs); quality of all reagents and materials were checked and handled according standard procedures. Known seropositive and negative sample was used as external quality control. All data collection procedure was under supervision.

#### Data analysis

After collection of all necessary information data entry and analysis was done using SPSS version 21.0 Statistical software. Variables were descriptively expressed using mean ± SD or number, percentage, and tables. Bivariate logistic regression analysis was conducted primarily to check association of each independent variable with the dependent variable. Odds ratio and 95% confidence interval was used to determine their level of significance and (P < 0.05) was considered as statistically significant.

### Result

#### Socio-demographic characteristics

In this study 460 study subjects were enrolled with a response rate of 100%. Majority of the study participants were males 265 (57.6%) with a mean age of 45.8 ± 11.8 likewise, majority of the study subjects were rural dwellers 274 (59.6%), 223 (50.7% are on the age group of (40–60) year (Table 1).

**Table 1:** Socio-demographic characteristics of study subjects (N = 460), North West Ethiopia, 2017

Characteristic	Number	Percent (%)
Gender		
Male	265	57.6
Female	195	42.4
Total	460	100
Age (in years)		
18–45	163	35.4
46–60	233	50.7
> 60	64	13.9
Residence		
Rural	274	59.6
Urban	186	40.4
Educational level		
Illiterate	140	30.4
Elementary school	168	36.5
High school	152	33

#### Seroprevalence and factors associated with hcv

Among the 460 study subjects, 77 (16.7%) were found to be serologically reactive for HCV. Higher proportion of HCV was found in females 39 (20%) and rural dwellers 63 (23%) as compared to their counterpart. On the other hand, highest percentage 64 (28%) of HCV) was detected in diabetic study subjects as compared to non diabetic study subjects 13 (6) Table 2).

**Table 2:** Bivariate logistic regression analysis of factors associated with seroprevalence of HCV among diabetic mellitus patients (N = 460) in North West Ethiopia, 2017

Variables	HCV-status, n (%)		Total n (%)	COR	95% CI	P-value
	No	Yes				
Gender						
Male	227 (85.7)	38 (14.3)	265 (100)	1		
Female	156 (80.0)	39 (20.0)	195 (100)	1.56	0.95–2.60	0.081
Age						
18–45	150 (92.0)	13 (8.0)	163 (100)	1		
46–60	176 (75.5)	57 (24.5)	233 (100)	0.268	0.1–0.5	< 0.01
> 60	57 (89.1)	7 (10.9)	64 (100)	0.7	0.3–1.8	0.481
Residence						
Rural	211 (77.0)	63 (23.0)	274 (100)	3.7	2.0–6.8	< 0.01
Urban	172 (92.5)	14 (7.5)	186 (100)	1		
Educational level						
Illiterate	98 (70.0)	42 (30.0)	140 (100)	0.4	0.2–0.7	< 0.01
Elementary school	142 (84.5)	26 (15.5)	168 (100)	2.9	1.3–6.4	< 0.01
High school	143 (94)	9 (6)	152 (100)	1		
History of active smoking						
Yes	24 (80)	6 (20)	30 (100)	1.3	0.5–3.2	0.612
No	359 (83.5)	71 (16.5)	430 (100)	1		
History of alcohol consumption						
Yes	125 (83.9)	24 (16.1)	149 (100)	0.9	0.5–1.6	0.782
No	258 (83.0)	53 (17.0)	311 (100)	1		
History of surgical procedure						
Yes	11 (64.7)	6 (35.3)	17 (100)	2.9	1.02–7.9	0.045
No	372 (84.0)	71 (16.0)	443 (100)	1		
History of hospitalization						
Yes	172 (77.8)	49 (22.2)	221 (100)	2.1	1.3–3.6	< 0.01
No	211 (88.3)	28 (11.7)	239 (100)			
History of blood transfusion						
Yes	98 (77.8)	28 (22.2)	126 (100)	1.7	0.9–2.8	0.055
No	285 (85.3)	49 (14.7)	334 (100)	1		
History of intravenous therapy						
Yes	146 (75)	49 (25)	195 (100)	2.8	1.7–4.7.0	< 0.01
No	237 (89.4)	28 (10.6)	265 (100)	1		
History of dental procedure						
Yes	62 (79.5)	16 (20.5)	78 (100)	1.4	0.7–2.5	0.331
No	321 (84)	61 (16)	382 (100)	1		
Uvulotomy						
Yes	360 (87)	54 (13)	414 (100)	0.15	0.08–0.3	< 0.01
No	23 (50)	23 (50)	46 (100)	1		
History of ear piercing						
Yes	189 (74.4)	65 (25.6)	254 (100)	5.6	2.9–10.6	< 0.01
No	194 (94.2)	12 (5.8)	206 (100)	1		
History of household blood contact						
Yes	51 (61.4)	32 (38.6)	83 (100)	4.6	2.7–7.9	<0.01
No	332 (88.1)	45 (11.9)	377 (100)	1		
History of circumcision						
Yes	190 (88)	26 (12)	216 (100)	0.5	0.3–0.8	< 0.01
No	193 (79)	51 (21)	244 (100)	1		
History of immunosuppressive disease						
Yes	58 (67.4)	28 (33.6)	86 (100)	3.2	1.9–5.5	< 0.01
No	325 (87)	49 (12.3)	374 (100)	1		
History of abortion						
Yes	58 (73.4)	21 (26.6)	79 (100)	2.1	1.2–3.7	< 0.01
No	325 (85)	56 (15)	381 (100)	1		
Diabetic mellitus						
Yes	166 (72)	64 (28)	230 (100)	6.4	3.4–12.0	< 0.01
No	217 (94)	13 (6)	230 (100)	1		
BMI						
< 18	158 (78.6)	43 (21.4)	201 (100)	0.5	0.3–0.85	< 0.01
18–24	215 (87.8)	30 (12.2)	245 (100)	1		
≥ 25	10 (71.4)	4 (28.6)	14 (100)	0.4	0.1–1.2	0.091
FBS						
< 126	272 (94.4)	16 (5.6)	288 (100)	1		
≥ 126	111 (64.5)	61 (35.5)	172 (100)	0.1	0.6–0.2	< 0.01

*COR* Crude Odds Ratio, *CI* confidence interval, *BMI* Body Mass Index, *FBS* fasting blood sugar

According to the bivariate logistic regression analysis of factors, 15 of the variables were found to be associated with seroprevalence of HCV including Residence (3.7 (2.0–6.8)); history of intravenous therapy (2.8 (1.7–4.7)) and ear précising history (5.6 (2.9–10.6)) (Table 2).

This study tried to observe the variables which have association with HCV antibody status in bivariate logistics regression analysis using multivariate logistic regression method. Accordingly only 3 variables have statistically significant association with HCV antibody status; Uvulotomy (12.4:3.5–18.3; P < 0.01); FBS (8.6:1.7–13.0; P < 0.01) (Table 3).

**Table 3:** Multivariate logistic regression analysis of factors associated with seroprevalence of HCV among diabetic mellitus patients (N = 460) in North West Ethiopia, 2017

Variables	HCV-status, n (%)		Total n (%)	AOR	95% CI	P-value
	No	Yes				
Age						
18–45	150 (92.0)	13 (8.0)	163 (100)	1		
46–60	176 (75.5)	57 (24.5)	233 (100)	0.4	0.07–1.78	0.213
> 60	57 (89.1)	7 (10.9)	64 (100)	1.1	0.06–23.9	0.931
Residence						
Rural	211 (77.0)	63 (23.0)	274 (100)	0.3	0.1–1.8	0.216
Urban	172 (92.5)	14 (7.5)	186 (100)	1		
Educational level						
Illiterate	98 (70.0)	42 (30.0)	140 (100)	0.68	0.1–4.3	0.681
Elementary school	142 (84.5)	26 (15.5)	168 (100)	0.6	0.1–2.9	0.557
High school	143 (94)	9 (6)	152 (100)	1		
History of hospitalization						
Yes	172 (77.8)	49 (22.2)	221 (100)	1.3	0.1–16.1	*0.843*
No	211 (88.3)	28 (11.7)	239 (100)			
History of intravenous therapy						
Yes	146 (75)	49 (25)	195 (100)	0.7	0.06–8.3	*0.814*
No	237 (89.4)	28 (10.6)	265 (100)	1		
Uvulotomy						
Yes	360 (87)	54 (13)	414 (100)	12.4	3.5–18.3	< 0.01*
No	23 (50)	23 (50)	46 (100)	1		
History of ear piercing						
Yes	189 (74.4)	65 (25.6)	254 (100)	0.3	0.1–1.1	*0.071*
No	194 (94.2)	12 (5.8)	206 (100)	1		
History of household blood contact						
Yes	51 (61.4)	32 (38.6)	83 (100)	1.5	0.4–5.8	*0.591*
No	332 (88.1)	45 (11.9)	377 (100)	1		
History of circumcision						
Yes	190 (88)	26 (12)	216 (100)	0.4	0.1–1.8	*0.312*
No	193 (79)	51 (21)	244 (100)	1		
History of immune suppressive disease						
Yes	58 (67.4)	28 (33.6)	86 (100)	0.1	0.03–0.5	*<* 0.01*
No	325 (87)	49 (12.3)	374 (100)	1		
History of abortion						
Yes	58 (73.4)	21 (26.6)	79 (100)	0.3	0.1–1.6	*0.193*
No	325 (85)	56 (15)	381 (100)	1		
Diabetic mellitus						
Yes	166 (72)	64 (28)	230 (100)	0.4	0.1–2.2	*0.323*
No	217 (94)	13 (6)	230 (100)	1		
BMI				0.7	0.3–1.6	*0.394*
< 18	158 (78.6)	43 (21.4)	201 (100)	0.6	0.4–2.7	*0.519*
18–24	215 (87.8)	30 (12.2)	245 (100)	1		
≥ 25	10 (71.4)	4 (28.6)	14 (100)	0.1	0.01–1.03	*0.053*
FBS						
< 126	272 (94.4)	*16 (5.6)*	288 (100)	1	1.7–13.0	< 0.01*
≥ 126	111 (64.5)	*61* (*35.5*)	*172* (*100*)	*8.6*		

Italic values indicate statistically significant variables at P < 0.05)

*AOR* adjusted odds ratio, *CI* confidence interval

* Statistically significant association

### Discussion

HCV induced auto immunity against pancreatic β-cell leads to diabetes because of molecular mimicry between HCV and β-cell antigens [18]. Currently many studies establish the association of HCV and DM in a number of clinical studies though conflicting results are reported and interestingly, some studies have also shown that the prevalence of HCV infection in diabetics is much higher compared with the normal population [20–22].

The overall seroprevalence HCV in the study subjects was 77 (16.7%) and the proportion HCV among diabetic study subjects and non diabetic control groups were found 64 (28%) vs 13 (6%) respectively. The prevalence of HCV among diabetic study subjects (28%) were found significantly higher as compared to the same study subjects in Ethiopia (9.9%) Yemen (10%), Multan (13.7%), China (6.8%) India (5.7%) [20, 21, 24–26]. The seroprevalence of HCV in control group (6%) is significantly higher as compared to global prevalence of HCV in general population (3%); Ethiopia (3.3%), China (2.6%) and Yemen (0%) Multan (4.9%) [20, 21, 24, 26, 27].

In this study Multivariate logistic regression analysis result shows that study subject with uvulotomy, previous History of immunosuppressive disease, and study subjects with abnormal fast blood glucose level (≥ 126 mg/dl) showed statistically significant association with anti HCV antibody sero status (Table 3). Accordingly seroprevalence of HCV is significantly higher in study subject with pervious history of immunosuppressive diseases as compared to their counterpart 33.6% vs 12.3% respectively. Similarly seroprevalence HCV were significantly higher in study subjects with abnormal fast blood glucose level (≥ 126 mg/dl) (35.5%) as compared to their counterpart (5.6%) and had 8.6 times more risk of acquiring HCV infection as compared to study subjects normal blood glucose level (< 126 mg/dl) (AOR: 8.6 (1.7–13)). This statistically significant higher prevalence of HCV in clients with bad FBS is in agreement with study conducted by Jodaoon et al. [28]. This may be the result of the contagious nature of HCV and repeated exposure for finger pricks, daily insulin injection immune comprise state of diabetic subjects. Even though it is not statistically significant study subjects’ previous history of house hold blood contact had 1.5 times more risk of HCV infection as compared to their counterpart.

## Limitations

The authors would like to forward the following limitations: Due to resource constrain we had not used additional confirmatory tests especially for participants who were positive by the screening test.

As conclusion, in our study we have observed that the overall seroprevalence of Hepatitis C virus in our study subjects were found higher as compared to study conducted in the same study groups in our country. In our study seroprevalence of Hepatitis C virus was slightly higher as compared to its global prevalence in general population. Study subject with previous History of immunosuppressive disease, Uvulotomy, and study subjects with abnormal fast blood glucose level had statistically significant association with anti HCV antibody sero status. Therefore health education should be given about infectious nature of HCV and individual with immunosuppressive disease and diabetic patients should also screen for their HCV status.

## Abbreviations

AOR: Adjusted Odds Ratio
BMI: Body Mass Index
CI: confidence interval
COR: Crude Odds Ratio
DM: diabetes mellitus
ELISA: enzyme-linked immunosorbent assay
FBS: fasting blood sugar
HCV: Hepatitis C virus
HIV: Human Immunodeficiency Virus
IR: insulin resistance
SOPs: standard operating procedures
T2D: type 2 diabetes
VCT: Voluntary Counseling Test
WHO: World Health Organization

## References

[1] Lauer GM, Walker BD (2001). Hepatitis C virus infection. N Engl J Med.

[2] Zimmet P, Alberti KG, Shaw J (2001). Global and societal implications of the diabetes epidemic. Nature.

[3] Global Burden of Viral Hepatitis. WHO report April, 2010.

[4] Zignego AL, Ferri C, Pileri SA, Caini P, Bianchi FB (2007). Extrahepatic manifestations of Hepatitis C Virus infection: a general overview and guidelines for a clinical approach. Dig Liver Dis..

[5] Cacoub P, Poynard T, Ghillani P, Charlotte F, Olivi, Piette J (1999). Extrahepatic manifestations of chronic hepatitis C. MULTIVIRC Group. Multidepartment virus C. Arthritis Rheum..

[6] Terilli RR, Cox AL (2013). Immunity and hepatitis C: a review. Curr HIV/AIDS Rep..

[7] Lee M, Yang H, Lu S, Jen C, You S, Wang L (2012). Chronic hepatitis C virus infection increases mortality from hepatic and extrahepatic diseases: a community-based long-term prospective study. J Infect Dis.

[8] Maasoumy B, Wedemeyer H (2012). Natural history of acute and chronic hepatitis C. Best Pract Res Clin Gastroenterol.

[9] Omland L, Jepsen P, Krarup H, Schønning K, Lind B, Kromann-Andersen H (2011). DANVIR Cohort Study; Increased mortality among persons infected with hepatitis C virus. Clin Gastroenterol Hepatol.

[10] El-Kamary S, Jhaveri R, Shardell M (2011). All-cause, liver-related, and non-liver-related mortality among HCV-infected individuals in the general US population. Clin Infect Dis.

[11] Alberti A, Vario A, Ferrari A, Pistis R (2005). Review article: chronic hepatitis C-natural history and cofactors. Aliment Pharmacol Ther.

[12] Asselah T, Rubbia-Brandt L, Marcellin P, Negro F (2006). Steatosis in chronic hepatitis C: why does it really matter?. Gut.

[13] Leandro G, Mangia A, Hui J, Fabris P, Rubbia-Brandt L, Colloredo G (2006). Relationship between steatosis, inflammation, and fibrosis in chronic hepatitis C: a meta-analysis of individual patient data. Gastroenterology.

[14] Hui JM, Sud A, Farrell GC, Bandara P, Byth K, Kench JG (2003). Insulin resistance is associated with chronic hepatitis C virus infection and fibrosis progression. Gastroenterology.

[15] White DL, Ratziu V, El-Serag HB (2008). Hepatitis C infection and risk of diabetes: a systematic review and meta-analysis. J Hepatol.

[16] Harris EH (2005). Elevated liver function tests in type 2 diabetes. Clin Diabetes.

[17] Huang JF, Dai CY, Yu ML, Hsieh MY, Chuang WL (2008). Abnormal liver function test predicts type 2 diabetes: a community-based prospective study. Diabetes Care..

[18] Honeyman MC, Stone NL, Harrison LC (1998). T-cell epitopes in type 1 diabetes autoantigen tyrosine phosphatase IA2: potential for mimicry with rotavirus and other envirommental agents. Mol Med.

[19] Laskus T, Radkowski M, Wang LF, Vargas H, Rakela J (1998). Search for hepatitis C virus extrahepatic replication sites in patients with acquired immunodeficiency syndrome: specific detection of negative-strand viral RNA in various tissues. Hepatology.

[20] Jadoon NA, Shahzad MA, Yaqoob R, Hussain M, Ali N (2010). Seroprevalence of hepatitis C in type 2 diabetes: evidence for a positive association. Virol J..

[21] Ali S, Abera S, Mihret A, Abebe T (2012). Association of hepatitis C virus infection with type II diabetes in Ethiopia: a hospital-based case–control study. Interdiscip Perspect Infect Dis.

[22] Aboud RS (2015). The relationship between hepatitis C virus infection and diabetes mellitus type 2. J Pharm Chem Biol Sci..

[23] Central Statistics Agency of Ethiopia (CSA); Census 2007, Tigray Region.

[24] Thabet HM, AL-Souraqy AM, Abbas AH, AL-hogary IQ, Anam EN, Noaman NS (2014). The relationship of Hepatitis C and B with diabetes of Yemeni patients. Asian Pac J Health Sci.

[25] Laloo Demitrost, Walke Prashant, Bhimo Thongam, Prasad Lallan, Ranabir Salam (2015). Seroprevalence of hepatitis C infection in type 2 diabetes mellitus. Indian J Endocrinol Metab..

[26] Chen HF, Li CY, Chen P, See TT, Lee HY (2006). Seroprevalence of hepatitis B and C in type 2 diabetic patients. JCMA..

[27] Lavanchy D (2009). The global burden of hepatitis C. Liver Int..

[28] Jadoon NA, Shahzad MA, Yaqoob R, Hussain M, Ali N (2010). Seroprevalence of hepatitis C in type 2 diabetes: evidence for a positive association. Virol J.

